# Fall injuries among patients in selected hospitals of nepal: A cross-sectional study

**DOI:** 10.1038/s41598-025-22368-6

**Published:** 2025-10-03

**Authors:** Bhagabati Sedain, Puspa Raj Pant, Dhanendra Veer Shakya, Kamala Lamichhane, Uddhav Sigdel, Yogendra Bahadur Gurung, Koustuv Dalal

**Affiliations:** 1 Tribhuvan University, Padmakanya Multiple Campus, Kathmandu, Nepal; 2 Nexus Institute of Research and Innovation (NIRI), Unit for Prevention and Lifesaving Activities (NPLSA), Kathmandu, Nepal; 3 Central Department of Population Studies, Tribhuvan University, Kritipur, Nepal; 4 School of Health Sciences, Division of Public Health Science, Mid Sweden University, Sundsvall, 851 70 Sweden

**Keywords:** Fall, Injuries, Hospital-based, Cross-sectional, Nepal, Risk factors, Health care

## Abstract

Fall injury statistics are scarce in low- and middle-income countries like Nepal. This study examines fall-related injuries and associated risk factors to address critical gaps in injury epidemiology and prevention. Data was collected from 18 August to 17 September 2023 from the emergency and inpatient departments of seven hospitals in Nepal, one from each province. A structured questionnaire, based on the WHO Guidelines, was used for data collection from patients or caregivers. Among 1,175 injury patients, 38.6% sustained nonfatal fall injuries. Males comprised 58.9% of cases. Most falls (93.6%) were unintentional, and 64.5% occurred at home. 32% of all fall injury patients were children under 15 years, and 17.7% were those aged 65 years or older. Farmers and students accounted for 58.5% of all falls. Injured limbs were most common, followed by head injuries (21.7%). Regression analysis revealed disparities in fall injuries associated with province, geography, place of residence, occupation groups, castes, and gender of the patients. Marital status and household income were not found to be significantly associated with falls. Males were more likely than females to receive immediate care after fall injuries. Falls occurring at home disproportionately affect children, the elderly, and those involved in agricultural occupations. The findings highlight the urgent need for implementing targeted interventions, particularly in domestic and rural settings, and addressing inequalities in injury treatment.

## Introduction

The incident of fall is defined as a situation in which a person inadvertently comes to rest on the ground, floor, or other lower level, excluding a person’s intentional change in position^1,2^. Depending upon the energy released or subsequent impact, the severity of fall injuries is determined. The spectrum of the outcomes of fall injuries is broad, encompassing minor, non-life-threatening injuries to paralysing spinal injuries and even death. Fall is the second leading cause of unintentional injury-related death^3^. Global Burden of Disease (GBD) estimated that falls accounted for approximately 696,000 deaths (9.2 deaths per 100,000) in 2017, with the total number of fall-related deaths rising steadily^4,5^. Worldwide, falls are a significant cause of severe injuries and hospitalizations, often leading to permanent disabilities and numerous minor injuries. Each year, 37.3 million falls are severe enough to require medical attention^2,3^. The World Report on Child Injury Prevention has also highlighted the public health importance of fall injuries in children, as they are the cause of more than half of all nonfatal injuries^6^. Children and older people are at higher risk of falling due to their vulnerable physical conditions. Similarly, falls are the tenth leading cause of Disability-Adjusted Life Years (DALYs) among people aged 75 years and older^4^. Falls are the most common type of injury, and an overwhelming majority (80%) occur in Low- and Middle-Income Countries (LMICs).

Falls occur in all age groups; however, the majority of the available reports^1,3^ and current published articles^5,7–9^ provide information on fall incidents involving older adults. Geographical locations and infrastructure designs are important contributing factors that can cause falls. Likewise, research has revealed that social and economic circumstances, including low income, lower educational level, employment status, inadequate housing, alcohol consumption, and risky behaviors, are the major risk factors for fall injuries^1,10^.

Analysis of GBD data from Nepal from 1990 to 2019 highlighted a rising trend in fall injuries^11^. Likewise, an analysis of Nepal Police records showed that more than 500 people die annually due to unintentional falls^12^. A household-based study found that among the injured respondents, 37.5% were due to falls^13^. Similarly, among the 184 Traumatic Spinal Cord Injury (TSCI) patients admitted between 2015 and 2016, it was found that falls accounted for nearly 70% of TSCI cases across all genders and age groups^3^. The latest estimates of the WHO showed a death rate of 8.7 per 100,000 from fall injuries, accounting for a total of 2,550 deaths in 2021^14^. These findings are based primarily on estimates, police records, or small geographical area-based studies. Despite their high number of incidents, fall injuries are under-recognized as a public health issue^1,2^. Thus, we gathered fall-related data from seven hub hospitals, one from each of the seven provinces of Nepal. We describe the current situation of nonfatal fall injuries in Nepal among the patients who attended the selected hospitals for treatment. This paper examines fall-related injuries and associated risk factors to address critical gaps in injury epidemiology and prevention.

## Methods

This paper analysed fall injuries from a cross-sectional study conducted among all injured patients attending the emergency and inpatient departments of seven hospitals. Data were collected from all patients with injuries available in the selected hospitals between 18-August and 17-September 2023. The hospitals were selected as the most preferred facilities for injury treatment and were purposely chosen to represent all seven provinces of Nepal (Table 1). The total number of beds in these hospitals was 2,550. A pretested, structured questionnaire was used to collect data from the patients or their caretakers in the hospitals. The questionnaire was prepared by adopting the WHO guidelines for conducting community surveys on injuries and violence^15^.

**Table 1:** List of study hospitals, locations, level, number of beds and their catchment areas.

SN	Hospital name and address	District/Province	Level	#Beds	Catchment area
1.	BP Koirala Institute of Health Sciences, Dharan	Sunsari/Province 1	Teaching Hospital	700	Eastern Nepal
2.	Madhesh Aca demy of Health Sciences, Dhanusha	Dhanusha/Province 2	Teaching Hospital	500	Madhesh Province
3.	National Trauma Center, Bir Hospital, Kathmandu	Kathmandu/Province 3	Specialty Hospital	200	National
4.	Gandaki Medical College	Chitwan/Province 4	Teaching Hospital	550	Pokhara
5.	Bheri Hospital	Banke/Province 5	Tertiary Zonal Hospital	150	Lumbini, Karnali, Sudurpaschim
6.	Karnali Academy of Health Sciences (KAHS)	Jumla/Province 6	Teaching Hospital	300	Karnali
7.	Mahakali Hospital, Kanchanpur	Kanchanpur/Province 7	Provincial Hospital	150	Sudurpaschim

The questionnaire was initially developed in the English language and subsequently translated into Nepali. The questionnaire consisted of three main sections: Basic Household and Patient Demographic Background, Types of Injuries and Mechanisms, Injury Treatment, and Costs Associated with Injury Treatment. The language consistency of the questionnaires was translated into and administered in the Nepali language. The research team developed a data collection tool, which was configured in the KoboCollect application on mobile devices.

A team of 12 trained field researchers undertook the task of collecting injury-related information from selected hospitals. The Research Assistants were trained to operate KoboCollect and provided orientation on the Data Collection tools. The Research Assistants entered the information into KoboCollect using their mobile phones, which were set up by the research team. The data collectors identified eligible patients with injuries through Emergency Department in-charge and traced any injury hospitalisation through registration of the inpatient department. All identified patients thus took part in the study. For severely injured patients or for children who were unable to respond, data were obtained from their caregivers. The study supervisors monitored the data collection process and consistency in data collection.

After collecting the data, it was sent online to the Central Office. Upon receiving and compiling all the data at the Central Office, it was saved in Excel software and then transferred to the SPSS software package. The data were then checked for consistency and entry errors using the necessary commands in SPSS software. Then, the necessary edits were made to the data for the final dataset. There were also some non-responses for a few questions. During the analysis of data from responses to such questions, cases with missing data were omitted. A manual for data collectors and supervisors was prepared in the Nepali language. This manual was also used for the orientation of the data collectors.

The study is meant to represent all patients who visited the seven study hospitals; therefore, we included patients attending the Emergency Department as well as the OPD during 1 month. Inclusion criteria: (1) all patients included regardless of age, severity, (2) patients attending Emergency as well as OPD, (3) patients of any injury, and (4) those willing and providing consent to participate. For severely injured patients, we sought consent and information from their caregivers.

While collecting data, multiple injury types were documented in the data file; the present analysis exclusively examined fall injury data. The demographic and socioeconomic characteristics, as well as other variables, including injury mechanisms and treatment costs, were used in the descriptive analysis. Categorical variables were presented using frequencies and percentages, while continuous variables were presented using means and standard deviations or median and interquartile range. We performed univariate and multivariable logistic regression analyses to assess the association between various factors and fall injuries. The multivariable analysis was used to adjust for potential confounders. Odds ratios (OR) were used to quantify the magnitude of the association between socioeconomic factors and falls. The socioeconomic variables, including province, rural/urban residence, Hill/Tarai residence, caste/ethnicity, age, sex, marital status, occupation, and monthly household income, were used in the analysis.

Statistical analysis was done using IBM SPSS Statistics version 29.0. A p-value of < 0.05 was considered statistically significant. This threshold identified factors significantly linked to fall injuries, providing evidence that the associations were not random.

## Results

Fall injuries were the most common cause of injury, accounting for nearly 39% (453) of all cases among the 1,175 patients interviewed across seven hospitals during the survey period. The highest proportion of fall injuries occurred among young adults aged 15–49 (41.3%), followed by individuals over 50 years (33.5%), and then among children aged 0–14 years (25.2%). Males constituted a larger proportion (58.9%) of the injured people compared to females (41.0%). The highest proportion of patients was currently married (40.5%). However, the number of unmarried was also significantly high (37.8%). A very high percentage (72.3%) of the fall injury patients had only primary education compared to a relatively small proportion (9.6%) of those with higher education (secondary, higher secondary, or bachelor’s degree). The most common occupations were farming/agriculture (30.4%) and 27.4% were students. A smaller proportion (5.7%) was engaged in skilled or professional occupations (Table 2).

**Table 2:** Distribution of fall injuries by sociodemographic characteristics among patients presenting to study hospitals during 18-August to 17-September 2023.

Characteristics	Percent (*n*)
Age Group (years)	
0–4	6.0 (27)
5–14	19.2 (87)
15–29	17.0 (77)
30–49	24.3 (110)
50–69	19.6 (89)
70+	13.9 (63)
Median (Inter quartile range)	36 years (50)
**Gender**	
Male	58.9 (267)
Female	41.0 (186)
**Marital status**	
Currently married	40.5 (117)
Ever married	21.7 (95)
Unmarried	37.8(165)
**Educational status**	
Primary (1–8 Grade)	73.2 (320)
Secondary (9–10 Grade)	13.7 (60)
Higher secondary (11–12 Grade)	10.3 (45)
Bachelor	2.7 (12)
**Type of occupation**	
Farmer	30.4 (133)
Self-employed	4.6 (20)
Skilled worker	5.7 (25)
Unskilled worker	4.1 (18)
Student	28.4 (124)
Unemployed	3.2 (14)
Other	23.6 (103)

Limbs were the most common site of injury following falls for both males and females (Table 3). Males who experienced fall injuries sustained injuries primarily to the upper limb (60.3%) and lower limb (42.0%), and then the neck and back region (7.1%). Females who experienced fall injuries sustained injuries primarily to the upper limb (52.7%) and lower limb (46.1%). Additionally, a significant proportion of injuries occurred to the neck and back parts of the body (12.1%). More females sustained injuries to their back (8.8%) compared to males (2.0%). Slightly over one-fifth of males (22.0%) and females (21.4%) sustained head injuries (Table 3).

**Table 3:** Percentage distribution of fall injuries by sex and affected body parts among the patients presenting to study hospitals during 18-August to 17-September 2023.

Parts of body injured	Male (*n* = 267)	Female (*n* = 186)
Head	22.0	21.4
Neck	5.1	3.3
Upper limbs	60.3	52.7
Ribs/chest	5.1	2.2
Abdomen	1.6	1.1
Back	2.0	8.8
Buttocks/hips	3.9	5.5
Genitalia	-	0.5
Lower limbs	42.0	46.1
Spine	4.7	2.2

*Note: Percentages of parts of the body injured include multiple responses.* .

The information on the location of falls showed that a higher proportion of falls occurred in the home environment (68%) compared to incidents outside the home (Table 4). Among males, a comparatively higher proportion of falls occurred outside the home (38%), while for females, over three-quarters (77%) occurred in the home. Approximately one in ten fall injury occurrences was reported on farms or fields. Workplace injuries accounted for 9% of falls among males, compared to only 4% for females, likely reflecting lower female participation in the labour force.

**Table 4:** Percentage distribution of injuries by places of falls and sex among patients presenting to the study hospitals during 18-August to 17-September 2023.

Place of injury occurrence	Male	Female	Total
Home	154 (62.9)	138(77.1)	292 (68.9)
Farm/fields	31 (12.7)	19(10.6)	50(11.8)
Roads	30 (12.2)	5(2.8)	35(8.3)
Leisure places	13(5.3)	6(3.4)	19(4.5)
Jungle/forest	9(3.7)	8 (4.5)	17(4.0)
Workplaces	22(9.0)	7(3.9)	29(6.8)
Others	8 (3.3)	3(1.7)	11(2.6)
Total	245(100.0)	179(100.0)	424(100.0)*

*Place of injury occurrence missing for 29 cases.

## Factors associated with fall injury

Logistic regression analysis (Table 5) revealed that Madhesh, Bagmati, Gandaki, and Lumbini all have significantly lower adjusted odds ratios, indicating that people in these provinces are much less likely to experience fall injuries compared to the reference group (Koshi province). In contrast, Karnali province shows a lower odds ratio; its association with fall injuries is likely not statistically significant after adjusting for other factors.

**Table 5:** Association between socioeconomic factors and fall-injuries among injured patients presenting to study hospitals between 18-August to 17-September 2023.

Explanatory variables	Unadjusted model		Adjusted model			
	COR	CI (95%)	AOR	CI (95%)	*p*-value	*N*(1,039)
Province						
Koshi (Ref.)	1.00		1.00			155
Madhesh	0.15***	0.094–0.245	0.17***	0.099–0.310	0.000	189
Bagmati	0.37***	0.233–0.586	0.29***	0.122–0.697	0.005	152
Gandaki	0.43***	0.282–0.654	0.38**	0.159–0.889	0.024	226
Lumbini	0.10***	0.059–0.160	0.11***	0.055–0.211	0.000	217
Karnali/Sudurpaschim	0.71	0.471–1.081	0.50*	0.252–1.006	0.053	236
Rural/urban residence						331
Rural (Ref.)	1.00		1.00		0.227	708
Urban	0.49***	0.374–0.635	0.80	0.578–1.120		
Hill/Tarai						631
Tarai (Ref.)	1.00		1.00		0.411	544
Hill	1.93***	1.521–2.448				
Caste/ethnicity						389
Hill Caste (Ref.)	1.00				0.748	240
Hill Janajati	1.02	0.738–1.406			0.334	54
Tarai Janajati	0.52**	0.282–0.950	1.00		0.967	103
Hill Dalits	1.19	0.772–1.843	0.17***	0.099–0.310	0.931	21
Madheshi Dalit	0.69	0.281–1.710	0.29***	0.122–0.697	0.067	198
Madheshi Castes	0.43***	0.299–0.626	0.38**	0.159–0.889	0.214	34
Muslim	0.29***	0.124–0.686	0.11***	0.055–0.211		331
Age group (years)			0.50*	0.252–1.006		
0–4 (Ref.)	1.00					43
5–14	0.89	0.440–1.794	1.00		0.262	145
15–29	0.15***	0.077–0.292	0.80	0.578–1.120	0.002	382
30–49	0.30***	0.155–0.578			0.031	328
50–69	0.53*	0.269–1.053	1.00		0.052	188
70+	1.44	0.665–3.099			0.754	89
Sex						
Female (Ref.)	1.00					422
Male	0.70***	0.546–0.889			0.021	753
Education			1.00			
Illiterate (Ref.)	1.00		0.17***	0.099–0.310		317
Primary	1.38*	0.943–2.026	0.29***	0.122–0.697	0.489	165
Secondary	0.62***	0.449–0.863	0.38**	0.159–0.889	0.782	276
Intermediate+	0.23***	0.159–0.329	0.11***	0.055–0.211	0.023	281
Marital status			0.50*	0.252–1.006		
Currently married (Ref.)	1.00					425
Widow/divorced/separated	1.58**	1.115–2.252	1.00		0.077	179
Never married	0.86	0.651–1.126	0.80	0.578–1.120	0.306	435
Main occupation						
Farmer (Ref.)	1.00		1.00			259
Self-employed	0.31***	0.175–0.535			0.054	82
Skilled worker	0.44***	0.257–0.748			0.144	79
Unskilled worker	0.46**	0.249–0.852			0.092	55
Student	0.64***	0.457–0.891			0.805	308
Unemployed	0.34***	0.176–0.657	1.00		0.207	53
Other	0.98	0.676–1.409	0.17***	0.099–0.310	0.497	203
Monthly income			0.29***	0.122–0.697		
Up to 20,000 (Ref.)	1.00		0.38**	0.159–0.889		306
20,001–30,000	0.73*	0.517–1.044	0.11***	0.055–0.211	0.208	224
30,001–40,000	0.82	0.566–1.199	0.50*	0.252–1.006	0.844	177
40,001–50,000	1.12	0.759–1.663			0.707	149
50,001–60,000	0.90	0.544–1.492	1.00		0.968	77
> 60,000	0.62 **	0.390–0.980	0.80	0.578–1.120	0.635	106

Note: Odds ratio is significant at 0.01 (***), 0.05 (**), and 0.1 (*) level; COR (Crude Odds Ratio) and AOR (Adjusted Odds Ratio).

Through the unadjusted analysis, we initially observed an inverse association between people living in urban areas and fall-related injuries (compared to those in rural areas). However, this association was found to be statistically insignificant after adjusting for other variables. The comparison between the hilly and Tarai regions also follows a similar trend. While the hilly region is initially associated with significantly higher odds of fall-related injury compared to the Tarai region, this association weakens upon adjusting for variables.

Among castes and ethnic groups, the Tarai Janajati, Madheshi, and Muslim population initially showed significantly lower odds of fall injuries compared to the Hill Caste group. However, after adjusting for confounding variables, only the Madheshi Caste group retains a borderline significant lower risk (AOR: 0.62, 95% CI: 0.375–1.022).

Compared to children aged 0–4 years, individuals in the 15–29 and 30–49 year age groups have a significantly lower risk of fall-related injuries, with adjusted odds ratios of 0.24 and 0.36, respectively, indicating high statistical significance. In contrast, the 5–14 and 70 + age groups do not differ significantly from the 0–4 year age group in terms of fall injury risk after accounting for other variables. Males showed significantly lower odds of sustaining fall-related injuries compared to females, even after adjusting for other sociodemographic factors (AOR: 0.69, 95% CI: 0.50–0.95).

We also found that individuals with intermediate or higher education were at a significantly reduced risk compared to illiterate individuals (AOR: 0.53, 95% CI: 0.30–0.93). Marital status and monthly income were not identified as significant factors in the adjusted model. In comparison to farmers, self-employed individuals were less likely to be affected by fall-related injuries, even after adjusting for other factors (AOR: 0.48, 95% CI: 0.24–0.97).

## Patient transportation to the hospital

Table 6 provides information on the time taken to transport injured patients to the hospital after the injury event, by gender. A higher proportion of male (52.9%) patients were taken to the hospital immediately after the event compared to females (40.7%). A substantial number of patients were taken to the hospital on the same day of the injury (70.7%). ‘Same day’ means they were taken to the hospital later in the day, but not immediately. For 29.3% of the patients, it took at least one day or a few days to see the doctor for their injuries.

**Table 6:** Percentage distribution of fall injury patients by hospital transport time and sex at study hospitals (18-August to 17-September 2023).

Time elapsed after	Male (*n* = 267)	Female (*n* = 186)	Total (*N* = 453)
Immediately	52.9	40.7	47.8
Same day	19.2	28.0	22.9
Next day	14.1	9.9	12.4
After a few days	13.7	21.4	16.9
Total	100.0	100.0	100.0

## Discussion

This study identified a total of 453 fall injury cases in seven selected tertiary hospitals over one month. The findings revealed that young to middle-aged individuals with lower levels of education, predominantly those involved in agricultural occupations or students, were the major population groups exposed to fall injuries.

Similar to our study results, the STEPS survey, conducted in 2019, also found that falls are an important cause of unintentional injuries^16^. The findings of this study showed that the prevalence of fall injuries among individuals aged 30–49 years was the highest (24.3%), with a median age of 36 years. Similarly, the age-related findings of this hospital-based study are consistent with the analysis of five years (2014–2019) of police records on fall injuries in Nepal, which reported an average age of 36 years^12^. Similar to our findings, a study from Qatar also reported that fall-related injuries were most common among respondents aged 20 to 34 years, with a median age of the patients at 36 years^17^. The age pattern analysis of fall injuries in high-income countries differed slightly from our findings; the number of fall incidents increases with age, and the older age population is at higher risk^18–21^. According to our results, fall injuries were more common among males (58.9%), a pattern also observed in a study conducted in South-Eastern Australia^20^. However, among older adults, the occurrence of fall injuries was comparatively higher in females^21–25^. These findings underscore the need for age- and sex-specific fall injury prevention initiatives.

Research studies found that higher education is associated with a lower risk of fall-related injuries^10,26,27^. The 2021 population census of Nepal showed that half of the population had a primary education, and one-fourth had a secondary education (grades 9–10)^28^. In this study, about three-fourths of individuals with fall injuries had primary education, and only 13.7% of the injured people had secondary education. The census results and this study’s findings indicated a high risk of fall injuries in Nepal. Higher prevalence of fall injury in the population with primary education could be attributed to limited awareness of safety measures, lower access to injury prevention resources, and engagement in higher-risk manual or physical labour. Thus, the fall injury prevention program needs to be developed to meet the specific needs of the target population groups.

Regarding occupational status, fall injuries^29,30^ are found to be higher among construction workers. In our context, approximately 60% of the population is engaged in agriculture as their primary occupation^31^. This study also found that among the fall injury patients interviewed, 30.4% reported their occupation as farmers, and 28.4% were students. These findings underscore the urgent need for fall injury prevention initiatives targeting individuals involved in agricultural activities. Additionally, it emphasized the importance of implementing fall injury prevention measures in academic institutions.

Among fall injury patients, the most commonly injured body parts are the hip, head, wrist, and ankle^24,32^. The result also found that the limbs and head injuries were significant. The body parts injured in fall injuries by gender did not reveal any significant differences. For both males and females, an almost equal proportion of body part injuries was found, with the majority having limb injuries and head injuries. Limb injuries can significantly impact mobility, leading to challenges in daily activities, chronic pain, or even permanent disability, which may require long-term rehabilitation.

This study identified the common locations of fall injuries, with the home environment being the most frequent (Male = 62.9% and Female = 77.1%), followed by farms/fields and roads. Timsina (2017) also reported that 56% of fall injuries occurred in the home environment in the United States^21^. Similarly, a Trauma Registry analysis from Qatar reported that 68.4% of females and 28.2% of males sustained fall injuries at home^20^. These findings highlight the need for targeted fall prevention strategies in home and agricultural settings.

Research findings have shown that mental health, age, gender, occupational status, and place of residence are associated with the risk of fall injuries^29,30,33^. In our study, logistic regression analysis revealed that rural and hill residents, as well as Hill caste groups, are more likely to experience fall injuries compared to urban residents, Tarai residents, and Tarai-based ethnic groups. Fall injury risk was higher among females, children under 15 years, illiterate individuals, those involved in agricultural occupations, and individuals with lower household incomes. After controlling for certain socioeconomic variables, significant factors influencing fall injuries included province, age, sex, and education, with individuals from Koshi province, younger children, females, and those with lower education levels having a higher likelihood of fall injuries.

From the result, it was also found that a significant proportion of patients, especially males, were taken to the hospital immediately after the injury, indicating a relatively prompt response. However, 29.3% of patients experienced delays of at least one day, suggesting that barriers such as limited transportation, geographic challenges, or a lack of awareness about the importance of timely care may be present. These delays may worsen injury outcomes, underscoring the need for enhanced emergency response systems and increased awareness about the importance of seeking prompt medical attention.

This study highlights the complex factors associated with fall injuries in Nepal. Geographic location, demographic characteristics, and socioeconomic factors were found to influence the risk of fall injuries significantly. Urban residents and individuals from the Tarai region were less likely to experience fall injuries than their rural and hill counterparts, respectively. Similarly, higher levels of education and non-agricultural occupations were associated with a reduced risk of falls. Staggering two-thirds of all falls occur in the home environment, which warrants preventive interventions, including environmental modification and enforcement of building codes and regulations. These findings also underscore the need for targeted prevention strategies, particularly for vulnerable population groups.

The major limitation of this study is that it was conducted in only seven hospitals, one from each province, over a period of one month. As a result, many fall injury cases during the study period may have been missed, and the findings do not capture seasonal variations or trends over a longer timeframe. Moreover, conducting research over longer durations and involving a larger number of hospitals would contribute to a more comprehensive understanding of the burden and patterns of fall injuries in Nepal.

## Conclusion

Based on the findings, fall injuries were most common among young to middle-aged individuals, particularly those with lower levels of education and engaged in agricultural or student occupations. Females experienced a higher burden of fall injuries in the home environment, while males were more often injured outside the home or at the workplace. Limb injuries were most prevalent, but a significant number of individuals, especially females, also sustained injuries to the back and head. Socioeconomic and geographic disparities were evident, with rural, hill-residing, and less educated individuals being more vulnerable to fall injuries. Improving access to nearby health facilities can help ensure timely treatment. Gender-specific barriers must be addressed to support women’s access to care. Findings highlighted the importance of targeting fall injury prevention efforts toward high-risk groups such as young children, females, the illiterate, and specific geographic regions.

## Data Availability

The data is available upon valid request from Dr. Bhagabati Sedain (*bssedhai@gmail.com)*.
